# Nanopore Sequencing Provides Rapid and Reliable Insight Into Microbial Profiles of Intensive Care Units

**DOI:** 10.3389/fpubh.2021.710985

**Published:** 2021-08-27

**Authors:** Guilherme Marcelino Viana de Siqueira, Felipe Marcelo Pereira-dos-Santos, Rafael Silva-Rocha, María-Eugenia Guazzaroni

**Affiliations:** ^1^Departamento de Biologia, Faculdade de Filosofia Ciências e Letras de Ribeirão Preto (FFCLRP-USP), Ribeirão Preto, Brazil; ^2^Departamento de Biologia Celular e Molecular e Bioagentes Patogênicos, Faculdade de Medicina de Ribeirão Preto (FMRP-USP), Ribeirão Preto, Brazil

**Keywords:** nanopore sequencing, illumina sequencing, 16S rRNA, environmental monitoring, intensive care units, healthcare-associated infections

## Abstract

Fast and accurate identification of pathogens is an essential task in healthcare settings. Second-generation sequencing platforms such as Illumina have greatly expanded the capacity with which different organisms can be detected in hospital samples, and third-generation nanopore-driven sequencing devices such as Oxford Nanopore's minION have recently emerged as ideal sequencing platforms for routine healthcare surveillance due to their long-read capacity and high portability. Despite its great potential, protocols and analysis pipelines for nanopore sequencing are still being extensively validated. In this work, we assess the ability of nanopore sequencing to provide reliable community profiles based on 16S rRNA sequencing in comparison to traditional Illumina platforms using samples collected from Intensive Care Units of a hospital in Brazil. While our results demonstrate that lower throughputs may be a shortcoming of the method in more complex samples, we show that the use of single-use Flongle flowcells in nanopore sequencing runs can provide insightful information on the community composition in healthcare settings.

## Introduction

Surveillance and control of organisms commonly found in the microbiome of surfaces surrounding patients in hospital settings are among the main public health challenges faced worldwide. Healthcare-associated infections (HAIs) are one of the leading causes of patient morbidity and mortality and are often associated with prolonged hospitalizations that weigh on health systems (1, 2). Moreover, HAI-related organisms have been shown to persist in environments and are strongly correlated with antimicrobial resistance (AMR), which imposes difficulties in treatment and contributes to the emergence of new multidrug resistant pathogens in healthcare settings (2).

Massive parallel sequencing technologies are important tools to aid monitoring of healthcare environments (3). While traditional pathogen detection methods usually involve laborious cultivation and biochemical assays, molecular characterization of organisms using specific biomarkers, such as the 16S rRNA gene, bypasses these steps, enabling the identification of poorly-represented or even unculturable pathogens at a much faster rate (3, 4).

The minION (Oxford Nanopore Technologies - ONT) is one of the latest installments of high-throughput third-generation sequencers available. This nanopore-driven device was introduced to the market in the mid-2010s, and quickly gained popularity as a tool for clinical and environmental monitoring and (meta)genomic profiling due to its capacity to generate extremely long reads while remaining highly portable and relatively cheaper than most standard sequencing technologies (5, 6). According to Oxford Nanopore, currently commercialized starters' packs allow yields in the range of 40 Gigabases at an initial cost of one thousand dollars.

Long reads are of particular importance for microbial community profiling, given that the 16S rRNA gene cannot be entirely sequenced by traditional second-generation platforms and the choice of which variable region(s) to amplify directly affects which genera can be detected with these machines (7). Moreover, the often time-consuming library preparation steps and longer sequencing runs hold back the widespread adoption of second-generation devices for routine applications in healthcare settings, where rapid results are critical (8).

Despite gaining traction, nanopore sequencing protocols and analysis pipelines are still being validated by the community at large, and many research groups have been thoroughly evaluating its performance against traditional (usually Illumina) platforms (9–12). In the present study, we investigated how nanopore-based long-read sequencing compares to Illumina sequencing for the study of complex microbial communities from hospital surfaces. In order to identify potentialities and limitations of nanopore sequencing we resequenced a set of samples collected in a previous study that used Illumina's MiSeq to explore the microbial diversity within Intensive care Units in a Brazilian hospital (13). Despite the difference in approaches, the results obtained in this study are comparable to the findings reported in the original publication and support the same conclusions. Thus, nanopore sequencing may be implemented as a fast and accurate methodology for tracking the distribution of harmful microbial species throughout healthcare facilities.

## Materials and Methods

### Samples Acquisition

The samples used in this study were collected by a collaborator in a hospital in the city of Ribeirão Preto in the state of São Paulo (Brazil) in 2018. The procedures used to acquire the samples are fully described in a previous publication of our group (13). In short, selected surfaces from Intensive Care Units (ICUs) and Neonatal Intensive Care Units (NICUs) were thoroughly streaked with sterile swabs premoistened with sterile Amies media for 2 min, after which the swabs were placed in sterile 15 mL conical centrifuge tubes containing an additional 1 mL of sterile Amies media and kept under refrigeration until DNA extraction was performed. Metagenomic DNA was extracted using the MoBio Powersoil DNA isolation kit, and samples were stored in a −80°C freezer until further processing (13).

### Library Preparation

In the present study, ~5 ng of the extracted DNA from these samples were used as template for barcoding PCR using primers provided in ONT SQK-16S024 sequencing kit. PCR amplifications were made with Phusion polymerase (NewEngland Biolabs) under the standard reaction protocol, including the use of 3% DMSO. After an initial denaturation step at 98°C for 2'30” the reaction cycles in the PCR program were set as follows: denaturation at 98°C for 15”, annealing at 52°C for 15”, extension at 72°C for 01'30”, with a final extension step of 72°C for 5' after the 35th cycle.

Success of 16S amplification was assessed in 0.8% agarose gels stained with SYBR-Safe^TM^ (Thermo Fisher Scientific). In order to avoid loss of material, we did not perform a purification step before pooling the amplicons together. Instead, samples that showed well-defined ≈1,500 bp bands were mixed at proportional volumes, starting with 5 μL for the strongest bands. The pools were then purified with AMPure XP beads (Beckman Coulter) and resuspended in 10 mM Tris-HCl pH 8.0 with 50 mM NaCl as recommended by ONT. Nanodrop^TM^ One (Thermo Fisher Scientific) was used to assess concentration of the purified pools. If needed, concentrations were adjusted to 10–20 ng/μL. Finally, 5 μL of each pool were mixed with 0.5 μL of the Rapid adapter (RAP) of the SQK-16S024 sequencing kit and incubated for 5 min at room temperature.

Library preparation, as well as Flongle flowcells priming and loading steps were then carried according to instructions described in the manufacturer's protocol (version: 16S_9086_v1_revI_14Aug2019). Sequencing runs lasted for up to 24 h and were performed in MinION model Mk1B (ONT).

### Long-Read Data Processing

FASTQ files were generated and demultiplexed concurrently to sequencing using Guppy basecaller (version 4.0.9) in the fast basecalling setting. After the runs ended, passed reads had barcode sequences removed using guppy_barcoder command line utility (ONT). NanoFilt (version 2.7.1) (14) was used to filter reads based on quality (QScore > 10) and size (1,350–1,650 bp), and suitable reads were finally aligned to the NCBI refseq 16S database using Minimap2 (version 2.17) (15). The alignment output was parsed in R (version 4.0.5) with the pafr package (version 0.0.2) (16). Only unique alignments with overlaps >1,000 bp were considered for further analysis.

### Comparative Data Analysis

In our previous work, the V4 region of the 16S rRNA gene was amplified from metagenomic DNA samples and sequenced in 2 × 300 bp Illumina MiSeq runs. QIIME version 1.9.1 was used to determine Operational Taxonomic Units (OTUs) after sequencing (13). Data from the previous publication was retrieved and used for comparison with nanopore-generated results. For all of the statistical analyses, only the samples sequenced with both methods were taken into consideration, and in the nanopore dataset, bacterial taxa assigned to less than five reads in each sample were not considered in the analysis.

Richness, diversity and dissimilarity analyses were carried in R (version 4.1.0) using methods implemented in the package Vegan (version 2.5.7) (17). In short, rarefaction curves for each nanopore-sequenced sample were obtained using the function *rarecurve()*, alpha-diversity for both datasets was calculated with the function *diversity()* using the Shannon method, and distance matrices used for hierarchical clustering were obtained with the *vegdist()* function using the Bray-Curtis method. Clusterization was performed with *hclust()* function using Ward's algorithm. Tanglegrams were plotted and the cophenetic correlation between dendrograms was calculated with the function *corr.dendlist()* from the dendextend package (version 1.15.1) (18). Permutational multivariate analysis of variance (PERMANOVA) tests were employed using the function *adonis2()* from Vegan to assess whether the microbiome composition in the different hospital sites and cleaning conditions differed using both sequencing methods. Determination of microbial biomarkers in ICU wards was performed using LEfSe command-line tool version 1.0 (19).

## Results

### Flongle-Powered Nanopore Sequencing Can Paint a Reliable Picture of the Community Composition of (N)ICUs Despite Its Limited Throughput

In a previous work from our group, samples from inanimate surfaces of (N)ICUs of a hospital in Brazil were collected and sequenced using an Illumina platform (MiSeq) after amplification of the ≈300 bp V4 region of the 16S ribosomal RNA gene (13). In the present study, samples from this original work were resequenced using minION, a nanopore-based platform, which allows full-length sequencing of the 16S gene (Supplementary Figure 1).

In total, we have resequenced 31 out of 43 samples collected in the previous work. Eighteen of the resequenced samples were collected in the ICU (either before or after cleaning) and 13 were samples taken in the Neonatal ICU. The minION sequencing runs generated 503 Mbp and 542 Mbp in passed reads, with a total number of passed reads ranging between 1,611 (sample Ventilator-ICUaA) and 34,083 reads (sample Pump-ICUa), averaging at 14,296 reads across our samples (Supplementary Table 1). For comparison, in our previous work, 4.94 Gbp were generated by Illumina's MiSeq platform, with an average read count per sample of 34,621.

In addition to this, our strict processing pipeline resulted in a massive removal of data. On average, 80% of reads did not meet our criteria of (i) having QScore > 10 (i.e., 90% of basecalling accuracy) and (ii) being as large as 1,650 bp but not smaller than 1,350 bp. Aside from having the highest number of raw reads, sample Pump-ICUa also had the highest loss (92.5%) of reads after processing.

As a direct consequence of the differences in throughput between sequencing platforms, estimates of diversity within samples in the Illumina dataset are generally greater than those of the nanopore (Supplementary Figure 2). Nonetheless, hierarchical clustering of the samples shows a high level of agreement at genus level between the two approaches, with correlation indexes ranging from 60.3 to 95.6% in different subsets of our samples (Supplementary Figure 3). Moreover, rarefaction curves show that the vast majority of the nanopore-sequenced samples had a number of counts that far exceeded the requirement for saturation even after processing (Supplementary Figure 4). These results seem to indicate that, even with a lower throughput, nanopore sequencing with Flongle flowcells was able to provide satisfactory amounts of data and has the potential to support subsequent community analysis.

### Both Sequencing Approaches Revealed Shortcomings of the Cleaning Protocols Employed to Sanitize ICU Surfaces

A main finding in our previous work was the apparent unevenness of efficacy of the concurrent cleaning procedures employed in the ICU wards. In fact, community composition analysis not only showed that the most abundant genera present in the samples collected before the cleaning also comprised the majority of the microbiota after the sanitation procedures took place, but that there was also a noticeable increase in the relative abundance of certain genera in the surfaces after cleaning (13).

**Figure 1:**
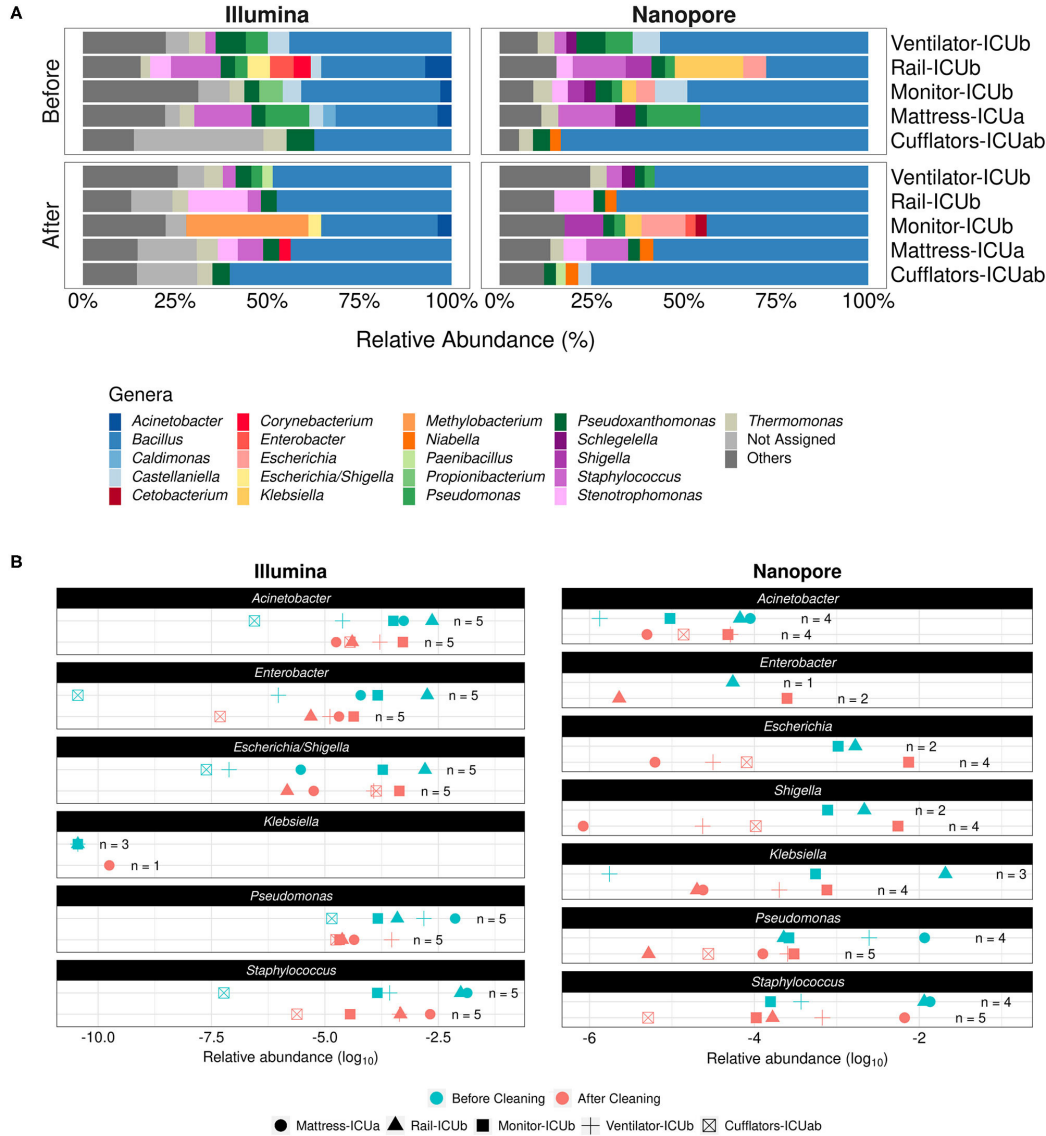
Comparison of the effect of cleaning routines in the ICU microbial profiles according to Illumina and Nanopore sequencing. **(A)** Stacked bar plots depicting main genera detected in ICU samples collected before and after concurrent cleaning with both approaches. Genera with relative abundances below 2.5% were grouped under the label “Others.” **(B)** Dot plots showing the relative abundances of specific HAI-related organisms in different samples (represented by different shapes in the plots) before (cyan) or after (red) cleaning, as detected by both sequencing methods. The number of samples in which each of the genera was found (*n*) is shown in the panels.

These conclusions can also be reached when assessing our nanopore sequencing dataset. For the comparison between relative abundances before and after cleaning, ten of the ICU samples that provided information to five different sites in both conditions, were assessed. Despite the differences in throughput and sensitivity between the two approaches, there is a great similarity between the most abundant bacterial genera detected in samples both with Illumina and nanopore sequencing, as shown in Figure 1A, and results indicate no statistical significance in the composition of the microbiota due to the sequencing method (PERMANOVA *pseudo* F-ratio = 1.52, *p*-value > 0.1). In the five sites, *Bacillus* was the predominant genus before cleaning (on average, 52% of relative abundance estimated with Nanopore and 34.9% with Illumina), followed by genera like *Staphylococcus* (11% with Nanopore vs. 10.6% with Illumina), *Stenotrophomonas* (4.36% with Nanopore vs. 5.6% with Illumina), *Pseudomonas* (6.8% with Nanopore vs. 7% with Illumina), *Pseudoxanthomonas* (4.75% with Nanopore vs. 5.49% with Illumina) and *Castellaniella* (8.1% with Nanopore vs. 4.35% with Illumina). Due to the high similarity between the 16S rRNA sequences of *Escherichia* and *Shigella*, taxonomy databases may report their relative abundances as a combined *Escherichia/Shigella* “genus” (20), but our direct alignment approach actually reports each genus independently based on how the read was mapped to the database, even though their sequences might be too similar for accurate discerning based on 16S alone (21). Before cleaning, analysis of nanopore data shows that *Shigella* was present at a relative abundance of 5.70%, while *Escherichia* accounted for 5.65%. In the Illumina dataset, *Escherichia/Shigella* was detected at 6.12% in relative abundance values.

Considering 2.5% of relative abundance as a cutoff to determine the most abundant genera in the samples, the genera *Klebsiella, Schlegelella*, and *Niabella* were not detected in the Illumina dataset while *Enterobacter, Proprionibacterium, Acinetobacter, Corynebacterium*, and *Caldimonas* were absent from the Nanopore dataset.

After cleaning, *Bacillus* remained the predominant genus in the samples (60% with Nanopore vs. 46.30% with Illumina), followed by *Stenotrophomonas* (8.41% with Nanopore vs. 10.8% with Illumina), *Staphylococcus* (7.7% with Nanopore vs. 4.6% with Illumina), *Pseudomonas* (2.8% with Nanopore vs. 2.94% with Illumina) and *Pseudoxanthomonas* (3% with Nanopore vs. 4.4% with Illumina). Moreover, in addition to the three genera mentioned above, *Castellaniella, Cetobacterium* and *Enterobacter* were not detected by Illumina after cleaning, whereas *Methylobacterium, Acinetobacter* and *Corynebacterium* were absent from the nanopore dataset. After cleaning, *Escherichia/Shigella* relative abundance dropped in the Illumina dataset, reaching 3.48%. However, the opposite was reported for genera *Escherichia* and *Shigella* when analyzed with the nanopore approach, with relative abundances of, respectively, 11.9 and 10.5% in the samples they were present. Nonetheless, multivariate analysis of variance analysis indicates that the community composition did not change significantly with regard to either sequencing method (PERMANOVA *pseudo* F-ratio = 1.39, *p*-value > 0.1).

Despite this general agreement between the two datasets, we notice that the prevalence of key genera associated with hospital infections across different ICU sites before and after cleaning varies greatly with the chosen method. As shown by Figure 1B, while it is clear that Illumina sequencing was able to detect the majority of investigated genera in all five sites, even if at lower relative abundances, there is a remarkable absence of *Klebsiella*, detected only at relative abundances of ≈10^−10^ in few samples. It should be noted that *Klebsiella pneumoniae* was the most abundant species (23.2%) from a total of 108 bacterial strains—distributed among 12 genera—that were detected by standard cultivation methods and Vitek 2 identification system in biological samples of hospitalized patients at the same period of the time of our sampling (Table 1; Supplementary Table 2). With Nanopore, on the other hand, we detected all of the genera in fewer sites, especially *Enterobacter*, but were able to identify *Klebsiella* both before and after cleaning.

**Table 1:** Detection of clinical pathogens by Illumina and Nanopore at genus-level and species-level.

**Genus-level assignment**	**Illumina**	**Nanopore**	**Species-level assignment**	**Illumina**	**Nanopore**	**Isolates**
*Acinetobacter*	39.8%	81.6%	*A. baumanii*	–	1.73%	1.85%
*Enterobacter*	12.2%	3.05%	*E. asburiae*	–	N.D.	1.85%
			*E. cloacae*	–	N.D.	0.93%
*Escherichia*	6.20%	11.9%	*E. coli*	–	1.87%	16.67%
*Klebsiella*	0.00872%	27.2%	*K. oxytoca*	–	2.50%	1.85%
			*K. pneumoniae*	–	26.3%	21.30%
*Pseudomonas*	11.9%	14.4%	*P. aeruginosa*	–	N.D.	8.33%
*Staphylococcus*	15.5%	21.6%	*S. aureus*	–	N.D.	9.26%
			*S. auricularis*	–	7.08%	0.93%
			*S. capitis*	–	7.71%	1.85%
			*S. epidermidis*	–	11.95%	12.04%
			*S. haemolyticus*	–	8.03%	1.85%
			*S. hominis*	–	9.59%	2.78%
			*S. warneri*	–	N.D.	2.78%

*The table shows the maximum percentage of detection (in terms of relative abundance) in the environmental samples for taxa retrieved from hospitalized patients during the time of the sampling. The percentage reported for the isolates considers an n = 108 samples, shown in more detail in Supplementary Table 2. N.D., Not detected*.

Overall, nanopore-generated estimates of the presence of pathogens in different samples were more conservative than those provided by Illumina. However, both approaches hint toward a lack of efficacy of the concurrent cleaning methods employed by hospital staff. Our results support that these practices do not seem to be adequate to sufficiently remove several HAI-related genera from inanimate surfaces and can end up serving as potential routes of cross-contamination of pathogens across hospital sites.

### Site-Specific Taxonomic Biomarkers Detected by Illumina and Nanopore Platforms Allow Spatial Monitoring of the Microbiota in ICU Wards

In addition to exploring the differences in microbial profiles as a result of the cleaning methods employed by ICU staff, the investigation of differences in the composition of the microbiota across sections of the hospital in our previous study could explain the prevalence of certain nosocomial diseases in these sites (13).

In consonance with the results presented in the previous section, there was no significative difference regarding the most abundant genera in the NICU samples when comparing the sequencing methods employed (PERMANOVA *pseudo* F-ratio = 1.97, *p*-value > 0.05). At a cutoff level of 2.5% in relative abundance, there were 32 genera detected with Illumina and 28 with Nanopore. The shared genera include common HAI-related organisms such as *Serratia, Bacillus, Delftia, Haemophilus, Stenotrophomonas, Acinetobacter, Streptococcus*, and *Staphylococcus*. Genera like *Aliterella, Pseudopropionibacterium, Alloiococcus, Klebsiella, Kingella, Cylindrospermum, Massilia, Enterococcus*, and *Aggregatibacter* were exclusive to the Nanopore dataset, whereas *Propionibacterium, Marinomonas, Clostridium_sensu_stricto, Tepidimonas, Lysobacter*, and *Lactobacillus* were present only in the Illumina dataset.

**Figure 2:**
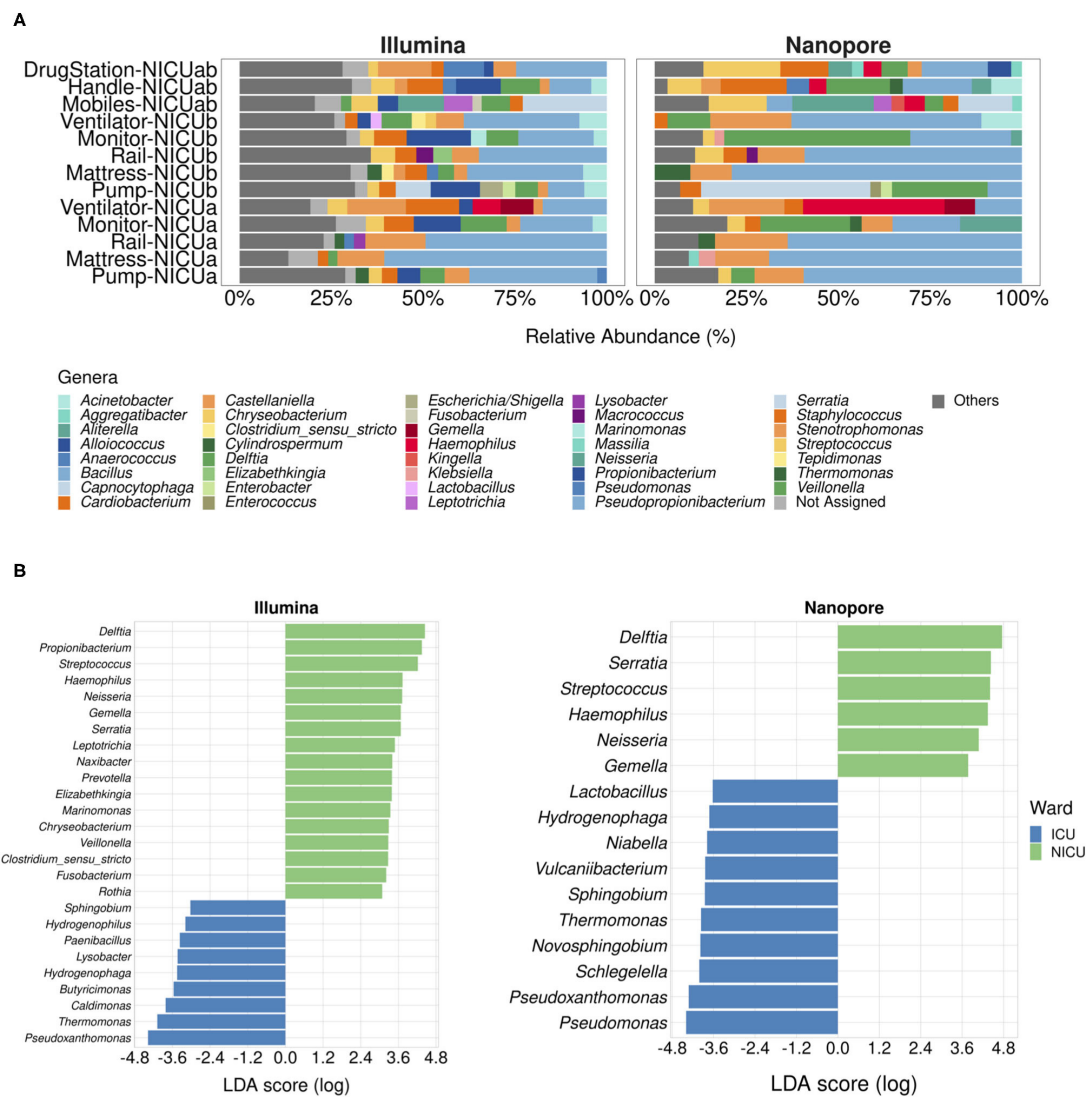
Neonatal Intensive Care Units microbial profiles obtained with Illumina and nanopore sequencing. **(A)** Stacked bar plots depicting main genera detected in NICU samples using both methodologies. Genera with relative abundances below 2.5% were grouped under the label “Others.” **(B)** Ward-specific taxonomic biomarkers for (N)ICU detected with linear discriminant analysis effect size (LEfSe). Statistical significance was defined as *p* < 0.05.

Nonetheless, we found the differences in relative abundance between Illumina and Nanopore to be more pronounced in the NICU samples than in the ICU samples. As seen in Figure 2A, main genera in the NICU include *Bacillus* (on average, 40.5% of relative abundance estimated with Nanopore and 28.8% with Illumina), *Capnocytophaga* (14.6% with Nanopore and 22.8% with Illumina), *Delftia* (18.58% with Nanopore and 7.49% with Illumina), *Neisseria* (14.3% Nanopore with and 12.5% with Illumina), *Stenotrophomonas* (12.3% with Nanopore and 9.29% with Illumina), *Haemophilus* (13.4% with Nanopore and 7.6% with Illumina), *Staphylococcus* (7.96% with Nanopore and 6.4% with Illumina) and *Pseudomonas* (6.12% with Nanopore and 5.07% with Illumina). In one sample, *Serratia* was detected at a relative abundance of 46% in the Nanopore dataset, while it appeared at 9.50% in the Illumina dataset.

Both in our previous work and in the present study, NICU was shown to be more diverse than ICU (Supplementary Figure 2) (13). With the 2.5% cutoff, an average of 26% of the genera present in NICU samples sequenced with Illumina fell under the label “Others”, highlighting the prevalence of low-abundance genera in these samples. In the Nanopore dataset, on the other hand, low-abundance genera within the samples comprised, on average, only 10% of the total diversity, leading to a possible overestimation of the most abundant genera.

In order to assess whether these differences impact the determination of taxonomic biomarkers in the samples, we employed an algorithm for high-dimensional biomarker discovery that can associate genera specifically associated to the different wards (19). Figure 2B shows which genera were found to be associated with (N)ICU wards using both sequencing datasets. As expected, nanopore sequencing retrieved fewer site-specific genera when compared to Illumina, however, all of the genera associated with NICU with higher LDA scores (apart from *Propionibacterium*) were equally found in both datasets, while ICU taxonomic biomarkers showed more variation. As a whole, these results reinforce that Nanopore's capacity to detect less abundant genera could have been undermined by the lower throughput of Flongle flowcells.

### Full-Length 16S rRNA Sequencing Provides Insight Into the Species-Level Composition of ICU Microbial Communities

One great advantage of generating reads that encompass the entire length of the 16S rRNA gene is that taxonomic assignment can be performed by direct mapping of the query reads to the 16S reference database, which ultimately may associate each read to a particular bacterial species (22).

**Figure 3:**
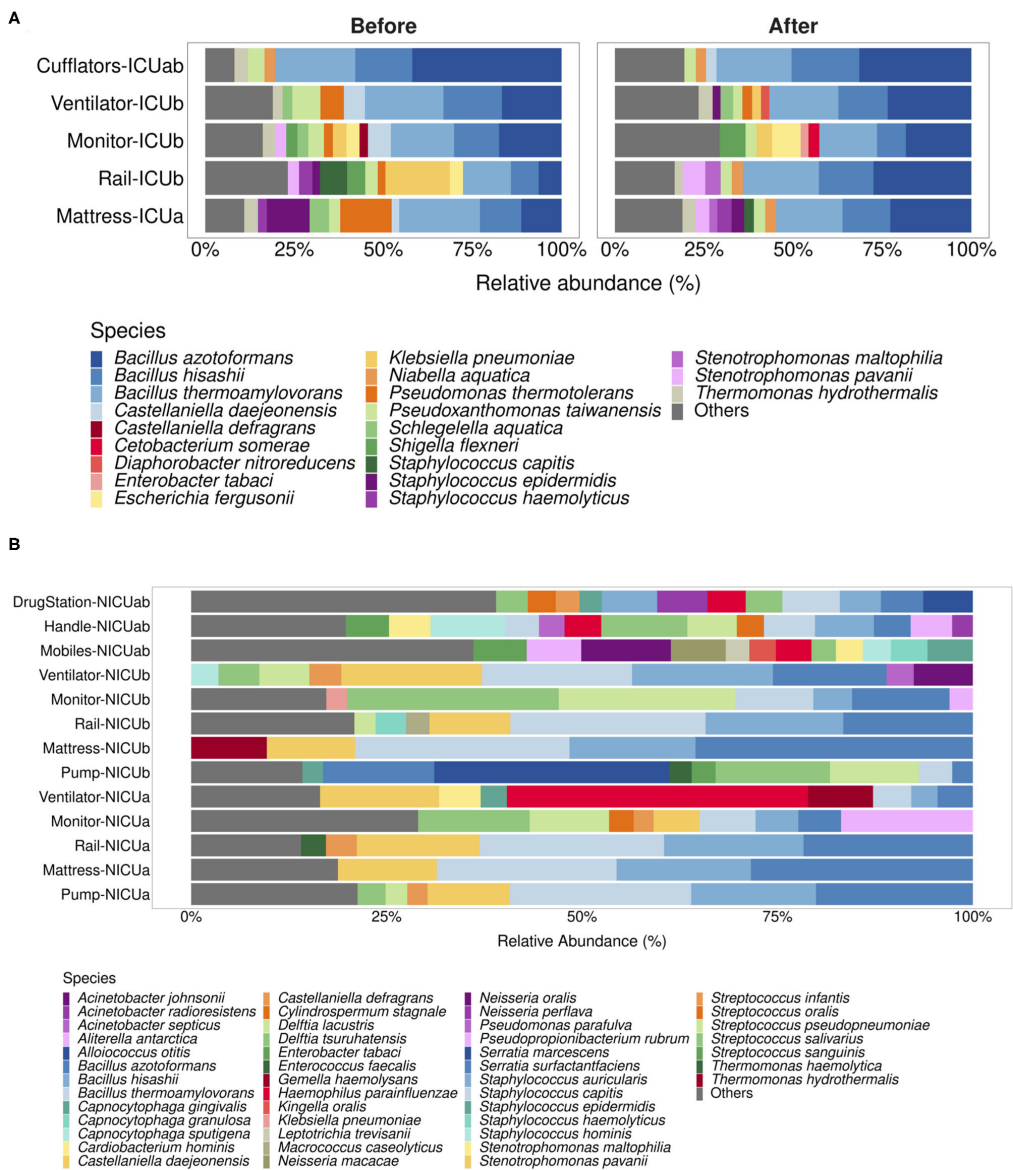
Species-level taxonomic assignment to (N)ICU samples. **(A,B)** depict the main species assigned to nanopore-generated reads for ICU and NICU samples, respectively. Species with relative abundances below 2% for ICU samples or 2.5% for NICU samples were grouped into the label “Others.”

While Nanopore sequencing methods may still lack accuracy to enable exact species-level correspondence between sequenced reads and organisms (9), a detailed look into the species assigned to our nanopore reads may show revealing features of the microbial communities and provide clues to how HAI-related organisms can be able to circumvent the cleaning procedures and underlying motions of cross-contamination between hospital areas. Figures 3A,B show the top assigned species to each sample, while Table 1 draws a comparison between groups detected in our analysis and the isolates retrieved from hospitalized patients, which allows us to better appreciate some the strengths and limitations of the method. Species-level taxonomic assignment shows that both in the isolates and in the environment, *K. pneumoniae* is the more abundant species of *Klebsiella*, followed by *K. oxytoca* in a much smaller proportion. Moreover, *Staphylococcus epidermidis* appears prominently among the staphylococci both in the ICU surfaces and in the clinical isolates, and several *Staphylococcus* spp. found in hospitalized patients were identified in our analysis. Nonetheless, there is a remarkable absence of clinically relevant groups in our Nanopore species-level classification such as *S. aureus, P. aeruginosa* and both representatives of *Enterobacter*. Given that they were detected at genus level, it remains unclear whether these specific taxa were not found in the environment or simply misclassified.

## Discussion

Nanopore sequencing has emerged with the potential to radically change the landscape of the -omics sciences, and researchers are still becoming acquainted to the full breadth of possibilities it offers. As the community grows, standardization and validation of wet lab protocols and computational pipelines are imperative and, while efforts in this direction have been making great strides, there is still much room for improvement (6, 23).

Our results indicate that throughput in nanopore sequencing runs was limited in comparison to the amount of data previously generated by Illumina. This observation might be related to the use of Flonge flowcells in our sequencing runs. Introduced to the market in 2019 as a cheaper alternative to the standard conventional minION flowcell for smaller sequencing experiments, single-use Flonge flowcells have up to 126 nanopores available for sequencing instead of the standard 2,048 present in the regular flowcell, and provides theoretical yields of up to 2.8 Gbp, according to the manufacturer. In practice, however, in the two runs that comprised this work there were 83 and 60 pores available for sequencing at the start of the experiments, respectively, and we only reached about 0.5 Gbp of sequenced material per run. Nonetheless, as Oxford Nanopore keeps upgrading sequencing chemistries and flowcell designs, it is possible that some of these performance issues might be improved in the near future and better results may be obtained in real-life settings.

Amplification biases are another frequent concern during library preparation in massive parallel sequencing experiments, regardless of the sequencing platform employed (24). In this study, we could amplify only 31 out of 42 selected samples using Oxford Nanopore's commercial set of primers and a standard PCR protocol, obtaining different degrees of success for each sample. Although it should be noted that the concentrations and qualities of our metagenomic templates were uneven, and that amplification could probably be achieved if reaction conditions were fine-tuned for each sample (which is ultimately impractical in a real-life setting), these inconsistent results hindered our ability to properly purify and quantify the amplicons and we believe that this is the most likely cause for the observed disparity in the number of reads obtained across some samples.

Literature shows that the use of Oxford Nanopore's commercial barcoding primers also potentially interfere with one's capacity to detect certain bacterial taxa in complex samples. *Corynebacterium, Pseudomonas* and *Bifidobacterium* have been reported to be some of the affected genera (9, 10, 25). In our experiments, while we were able to detect *Pseudomonas* in relative abundances comparable to those previously observed in the Illumina experiments, *Corynebacterium* was indeed severely underrepresented. Moreover, we could not detect *Proprionibacterium* in our Nanopore dataset, even though it was detected as a NICU biomarker by Illumina sequencing. Approaches such as the design of custom primers have been proved to be effective in circumventing amplification limitations and providing more accurate community profiles with nanopore sequencing efforts (25). Moreover, one must also consider that differences in the databases used for taxonomic classification may also serve as a source of variability when drawing comparisons such as the ones in this study (20, 26).

Aside from amplification issues, the low number of usable reads after processing steps was another striking result we observed. Literature suggests that this is not uncommon in nanopore sequencing studies. The relatively high error rate of current basecalling algorithms is a known limitation of the method, and researchers often have to define arbitrary quality thresholds in order to balance reliability of information with loss of data. Moreover, size range cutoffs chosen to filter reads certainly have an impact on the number of reads retrieved in a given experiment. Thus, most reports for microbial community analysis using 16S we found applied different Q Score thresholds (usually Q Score > 7) and variable cutoff values (e.g., 1,300–1,950 bp, <1,700 bp, 1,400–1,700 bp, and 1,350–1,650 bp) (9, 10, 25, 27).

Despite the variability in experiment design choices, there is a general consensus that nanopore sequencing is robust enough to provide reliable community composition information, at least at genus level. Our results are favorable to this conclusion. Even though the differences in throughput led to different diversity estimates between Nanopore and Illumina datasets, the main genera detected in the samples were, with few noted exceptions, the same with both approaches and relative abundances were mostly comparable. For instance, Table 1 reports that the genus *Acinetobacter* was reported as present in a sample (Ventilator in ICUa after cleaning) with a staggering relative abundance of 81.6%. While this was also the sample in which this genus was detected with the highest relative abundance with Illumina (39.8%), such an overestimated value might be due to the fact that Ventilator-ICUaA was one of the samples with the least amount of reads after processing and reported only six different genera after taxonomic assignment. In this case, while the extracted information is the same (that is, *Acinetobacter* was reported at its maximum relative abundance in this specific sample both with Illumina and nanopore), the depth of sequencing may have played a role distorting the observed results, and normalization methods that better handle this sort of sparse data could help mitigating some of these aberrations (28).

Aside from assessing nanopore by comparison with Illumina, our analysis also revealed possible shortcomings of the previous Illumina sequencing effort itself. Aside from the remarkable observation that the appreciable detection of *Klebsiella* was only possible with nanopore sequencing, even though this genus was found to be an important pathogen in the hospital at that time, the absence of *Methylobacterium*, a known laboratory contaminant (29, 30), in our nanopore dataset suggests that the high abundances of this genus previously reported in the sample monitor-ICUb was due to contamination during sample processing.

While promising, 16S-based species-level microbial profiling using nanopore remains a rather controversial topic. Current error rates reported for long-read basecalling algorithms are usually higher than the differences between closely related species, which makes species-level assignment unadvisable due to the possibility of false positives (9, 10). Using a mock community, Winand and collaborators reported that, even though genus-level classification of nanopore is highly accurate, the use of absolute number of reads as a “tiebreak” between organisms of the same genus would not reflect the real composition of the community for all present species (9). Other works are more favorable to species-level taxonomic assignment using nanopore, arguing that low discrimination of closely related species (e.g., members of the genera *Bacillus* and *Escherichia*) is a limitation of using 16S as a biomarker by itself, and could be overcome if more comprehensive sequences, such as a larger region of the *rrn* operon (16S rRNA-ITS-23S rRNA), were employed instead (22, 25).

Upon investigation, we found that species-level taxonomic assignment to our nanopore reads indeed resulted in split categorization between different species within the represented genera. Reads belonging to the genus *Bacillus*, for instance, were assigned to *Bacillus azotoformans, Bacillus hisahii* and *Bacillus thermoamylovorans* in most samples. Without any additional information, it would be impossible to determine which of these species were actually present in the environments and their actual relative abundances. However, we believe that some of the results, although inconclusive, may direct further investigation. For instance, it has caught our attention that the most abundant *Escherichia* species in the ICU samples was *E. fergusonii*. In fact, in many samples it has been the sole *Escherichia* detected. While still considered a novel pathogen, a recent publication reported the presence of extended-spectrum β-lactamase-producing *E. fergusonii* in poultry farms in the state of São Paulo (31), which stresses the importance of monitoring this microorganism in healthcare facilities of our region. In the NICU samples, the knowledge that *Haemophilus parainfluenzae* is the sole representative of its genus in samples like ventilators might be an important clue to support accurate diagnostics of *Haemophilus* spp. nosocomial infections, since traditional phenotypic assays can lead to faulty results (32).

Moreover, environments such as NICUs are known to house several related species concurrently, and finding ways to accurately detect different related bacteria at species- or even strain-level is important for effective surveillance and diagnosis in healthcare settings (33, 34). In fact, as seen in Table 1 and Supplementary Table 2, a lot of subtlety about the prevalence of specific pathogens found in the clinical isolates we had information about would be lost at genus-level classification. However, the absence of key taxa in our nanopore dataset raises questions of whether they were not found in the environment or not properly assigned. In this regard, recently developed tools like the Bonito basecaller promise to take on some of nanopore's main limitations and may turn high-accuracy long-read sequencing into a reality within the next years (35).

In conclusion, it is exciting to us that a small team and a couple of days' worth of bench work were enough to verify findings that were previously obtained by a much larger cohort of our colleagues, all without the cumbersome requirement of sending samples back and forth to special facilities. As methods become more refined with time, nanopore sequencing certainly has the potential to empower healthcare settings to monitor environmental threats rapidly and reliably on a day-to-day basis, revolutionizing healthcare as we know it today.

## Data Availability Statement

The nucleotide sequences obtained in the present study have been deposited in the Sequenced Read Archive (SRA) database under the accession number PRJNA728896. A GitHub repository containing the scripts that we have developed for automatization of our pipeline, as well as reproducible code and examples on how our analysis was carried, can be found at https://github.com/GuazzaroniLab/nanopore_ICU_profiling.

## Author Contributions

M-EG and RS-R contributed to conception and design of the study. GS performed the wet-lab procedures, including 16S amplification and nanopore sequencing, conducted the data analysis, and wrote the first draft of the manuscript. GS and FP-d-S wrote the scripts for processing raw long-read data. All authors contributed to the article and approved the submitted version.

## Conflict of Interest

The authors declare that the research was conducted in the absence of any commercial or financial relationships that could be construed as a potential conflict of interest.

## Publisher's Note

All claims expressed in this article are solely those of the authors and do not necessarily represent those of their affiliated organizations, or those of the publisher, the editors and the reviewers. Any product that may be evaluated in this article, or claim that may be made by its manufacturer, is not guaranteed or endorsed by the publisher.
