# Supplementary material for: Nanopore Sequencing Provides Rapid and Reliable Insight Into Microbial Profiles of Intensive Care Units
Source: Front Public Health. 2021 Aug 27;9:710985. doi: 10.3389/fpubh.2021.710985 (PMC8429932; doi:10.3389/fpubh.2021.710985)
Supplement: Supplementary file 1 [file Data_Sheet_1.PDF]

Nanopore sequencing provides rapid and reliable insight into microbial profiles of Intensive Care Units

Guilherme Marcelino Viana de Siqueira[1], Felipe Marcelo Pereira-dos-Santos[2], Rafael Silva-Rocha[2†], María-Eugenia Guazzaroni[1†*]

[1]Faculdade de Filosofia Ciências e Letras de Ribeirão Preto (FFCLRP-USP), Ribeirão Preto, SP, Brasil. 14040-901
[2]Faculdade de Medicina de Ribeirão Preto (FMRP-USP), Ribeirão Preto, SP, Brasil. 14049-900
[†]These authors share last authorship

*** Correspondence:**
María-Eugenia Guazzaroni
meguazzaroni@ffclrp.usp.br

Supplementary Material

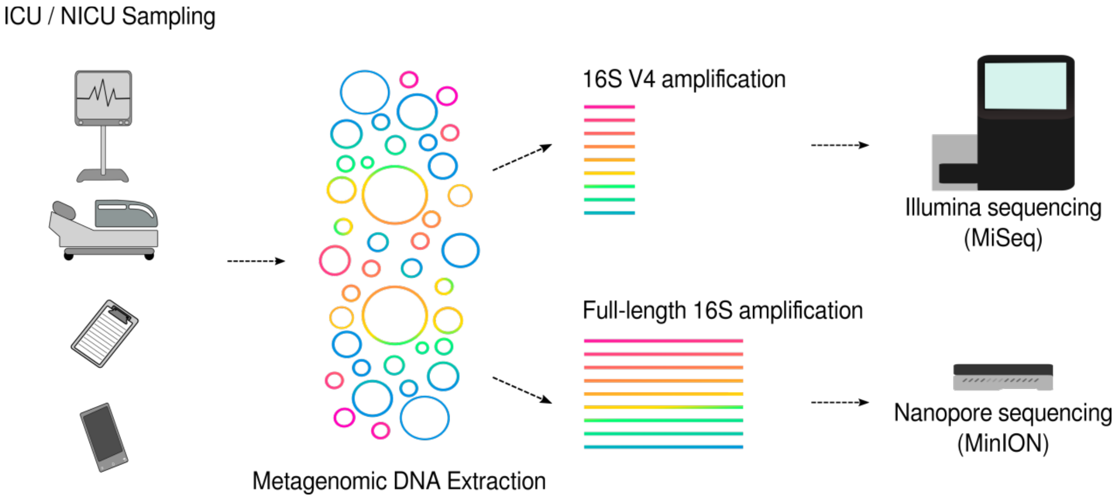

Figure S1. Overview of our experimental design. In a previous work of our group. metagenomic DNA was extracted from surfaces and objects surrounding patients in (N)ICUs of a hospital in Brazil. Samples were then subjected to V4 16S sequencing using Illumina MiSeq for microbial community profiling. In the present study, full-length 16S nanopore sequencing was performed in a subset of these samples, allowing us to compare both methods. For consistency, sample names followed the same code used in our previous work: sample source (e.g., "Handles" for door handles, "Ventilators", "Mobiles" for staff cell phone devices, "Monitors" for cardiac monitors...), followed by either ICU or NICU and the ward (a, b or ab). For concurrent cleaning analysis, an uppercase "A" identifies samples collected after the cleaning. A full description of the samples can be found in our previous publication (1).

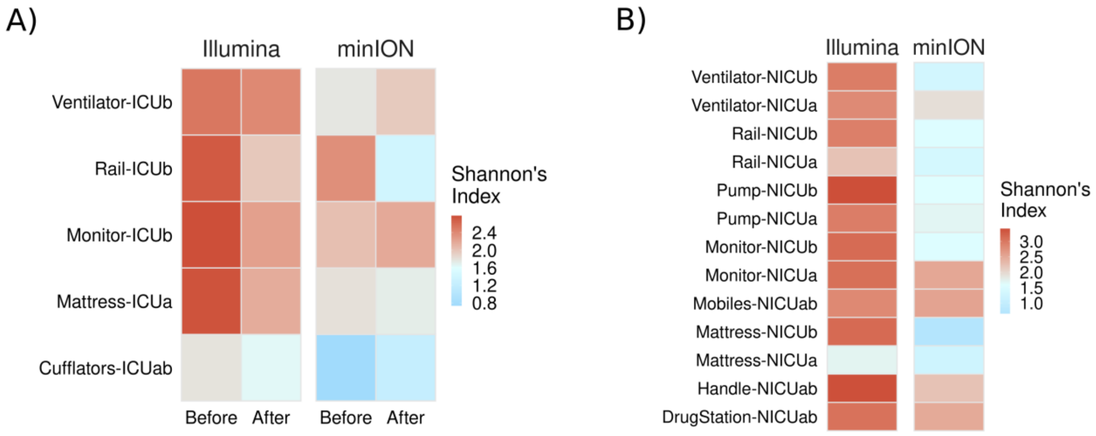

Figure S2 - Alpha diversity (Shannon Index) at genus level measured for samples from the nanopore and Illumina datasets. **A)** ICU samples before and after cleaning and **B)** NICU samples.

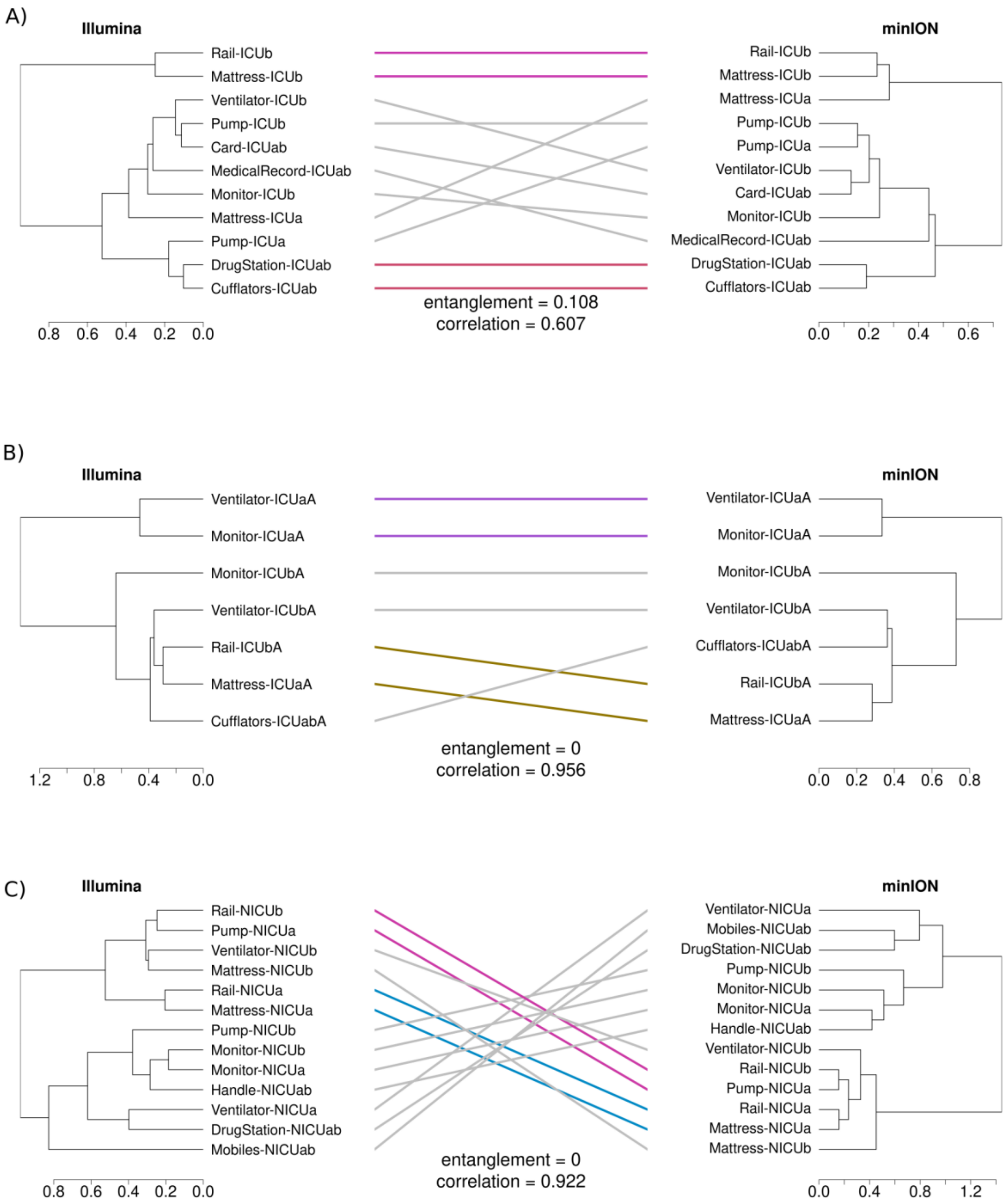

Figure S3 - Tanglegrams showing the hierarchical clustering (Ward's algorithm) similarities between samples sequenced with Illumina and nanopore in **A)** ICU samples before the concurrent cleaning, **B)** ICU samples after the concurrent cleaning, and **C)** NICU samples. Distance between groups was measured using Bray-Curtis index. Correlation and entanglement methods employed were, respectively cophenetic and "step2side". Colored lines indicate shared leaves between both dendrograms and horizontal rulers indicate the clustering heights.

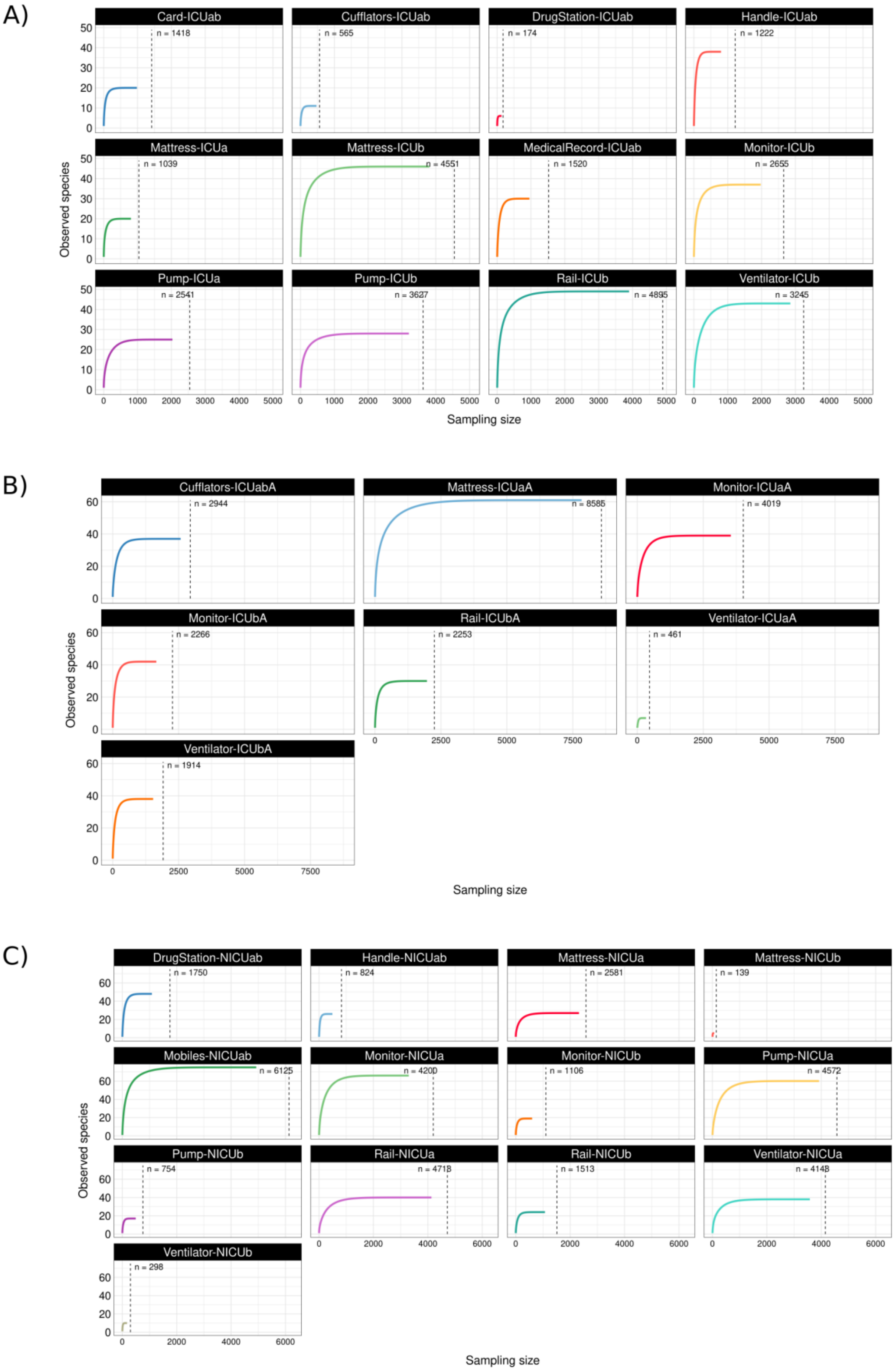

Figure S4 - Rarefaction curves for nanopore-sequenced samples. **A)** samples from ICU before the cleaning, **B)** samples from ICU after cleaning, **C)** samples from NICU. The dashed line demarcates the *n* number of reads that remained after all processing steps.

Table S1. Summary statistics of nanopore sequencing data for (N)ICU surface samples during different steps in our analysis pipeline. The total reads column refers to the number of reads effectively used at the taxonomic assignment step. For comparison, all of the samples were rarefied to a total of 33.708 reads prior to OTU clustering in the Illumina dataset analysis.

	after basecalling				after filtering and QC			
Sample	max (bp)	min (bp)	avg (bp)	total reads	max (bp)	min (bp)	avg (bp)	total reads
Card-ICUab	4226	36	1450.709	17317	1569	1351	1439.334	1418
Cufflators-ICUab	4081	51	1451.937	7174	1559	1355	1442.184	565
Cufflators-ICUabA	3159	71	1446.591	10538	1621	1352	1448.104	2944
DrugStation-ICUab	3140	75	1460.719	2152	1534	1352	1446.408	174
DrugStation-NICUab	3112	59	1444.486	6102	1597	1355	1444.446	1750
Handle-NICUab	3024	53	1436.484	2882	1565	1350	1439.511	824
Mattress-ICUa	3273	32	1450.524	12027	1619	1356	1442.259	1039
Mattress-ICUaA	3865	10	1431.443	31276	1581	1350	1448.602	8585
Mattress-ICUb	3119	41	1440.604	15215	1646	1351	1446.253	4551
Mattress-NICUa	3089	81	1446.941	8863	1638	1350	1448.1	2581
Mattress-NICUb	3128	50	1464.799	1635	1602	1353	1445.525	139
MedicalRecord-ICUab	3203	44	1402.943	19358	1626	1350	1436.101	1520
Mobiles-NICUab	3214	52	1434.405	21184	1629	1350	1440.422	6125
Monitor-ICUaA	3158	66	1444.956	14770	1637	1354	1444.867	4019
Monitor-ICUb	3106	53	1440.017	8925	1595	1350	1443.119	2655
Monitor-ICUbA	3308	36	1405.712	27027	1608	1350	1439.851	2266
Monitor-NICUa	15467	16	1427.924	14335	1595	1350	1432.931	4200
Monitor-NICUb	3207	30	1379.87	13543	1581	1350	1434.558	1106
Pump-ICUa	4113	24	1391.855	34083	1587	1350	1441.986	2541
Pump-ICUb	3182	10	1451.5	11899	1601	1354	1453.717	3627
Pump-NICUa	3211	54	1417.056	16759	1617	1350	1446.297	4572
Pump-NICUb	3174	59	1440.717	8088	1553	1353	1439.45	754
Rail-ICUb	3227	55	1423.863	16891	1598	1351	1442.948	4895
Rail-ICUbA	3251	36	1430.81	27337	1590	1350	1448.805	2253
Rail-NICUa	3195	58	1417.085	17144	1616	1350	1445.584	4718
Rail-NICUb	4720	24	1397.072	19717	1610	1354	1441.449	1513
Ventilator-ICUaA	3158	80	1448.31	1611	1588	1376	1448.026	461
Ventilator-ICUb	3147	52	1443.374	10911	1626	1351	1446.121	3245
Ventilator-ICUbA	3393	24	1408.932	25273	1575	1352	1437.071	1914
Ventilator-NICUa	3200	54	1442.009	14947	1604	1350	1449.432	4148
Ventilator-NICUb	4782	43	1454.293	3735	1566	1355	1439.678	298

Table S2. Isolates recovered from patients hospitalized in ICUs at the Hospital das Clínicas de Ribeirão Preto during the time period in which the samples used in this study were collected.

Species	Number of isolates	Relative percentage
Acinetobacter baumannii	2	1.85%
Burkholderia cepacia	1	0.93%
Cupriavidus pauculus	1	0.93%
Enterobacter asburiae	2	1.85%
Enterobacter cloacae	1	0.93%
Enterococcus faecalis	2	1.85%
Enterococcus faecium	1	0.93%
Escherichia coli	18	16.67%
Klebsiella oxytoca	2	1.85%
Klebsiella pneumoniae	23	21.3%
Morganella morganii	2	1.85%
Pseudomonas aeruginosa	9	8.33%
Ralstonia mannitolilytica	4	3.7%
Staphylococcus aureus	10	9.26%
Staphylococcus auricularis	1	0.93%
Staphylococcus capitis	2	1.85%
Staphylococcus epidermidis	13	12.04%
Staphylococcus haemolyticus	2	1.85%
Staphylococcus hominis	3	2.78%
Staphylococcus warneri	3	2.78%
Stenotrophomonas maltophilia	4	3.7%
Streptococcus gallolyticus	2	1.85%

References

1. Ribeiro LF, Lopes EM, Kishi LT, Ribeiro LFC, Menegueti MG, Gaspar GG, Silva-Rocha R, Guazzaroni ME. Microbial community profiling in intensive care units expose limitations in current sanitary standards. Front Public Heal (2019) 7:1–14. doi:10.3389/fpubh.2019.00240
